# Validation of the Hebrew version of the Community Assessment of Psychic Experiences in a sample of Israeli Hebrew speakers

**DOI:** 10.3389/fpsyt.2025.1548310

**Published:** 2025-05-14

**Authors:** Laurina Fazioli, Ahmad Abu-Akel, Bat-Sheva Hadad, Amit Yashar

**Affiliations:** ^1^ Department of Special Education, University of Haifa, Haifa, Israel; ^2^ School of Psychological Sciences, University of Haifa, Haifa, Israel; ^3^ The Haifa Brain and Behavior Hub (HBBH), University of Haifa, Haifa, Israel; ^4^ The Edmond J. Safra Brain Research Center for the Study of Learning Disabilities, University of Haifa, Haifa, Israel

**Keywords:** CAPE-42, cross-validation, Hebrew, psychometric properties, psychosis

## Abstract

**Introduction:**

The Community Assessment of Psychic Experiences (CAPE-42) is a reliable tool to assess psychotic experiences (PEs) in clinical and non-clinical populations, in research and clinical settings.

**Methods:**

To investigate cultural differences in PEs and control for pathological behavior in non-clinical groups, we developed a Hebrew version of the CAPE-42 using the translation/back-translation method. A total of 359 Hebrew speaking Israelis participated in an online study comprising the CAPE-42, the Autistic Quotient (AQ), the Center for Epidemiological Study – Depression Scale (CES-D), and the Prodromal Questionnaire – Brief Version (PQ-B). We examined the psychometric properties of the Hebrew CAPE-42—including its factor structure, internal consistency, gender invariance, and validity. We also investigated the independent and interaction effects of psychotic and autistic traits on depressive symptoms.

**Results:**

Reliability analysis demonstrated very good internal consistency, and confirmatory factor analysis supported the eight-factor model, which included depressive, social withdrawal, affective flattening, avolition, bizarre experiences, perceptual abnormalities, persecutory ideation, and magical thinking. Demonstrating its predictive and convergent validity, we found significant correlations with the CES-D and the PQ-B. The predictive model showed that both psychotic and autistic traits are independent, non-interacting, predictors of depressive symptoms.

**Conclusions:**

The Hebrew CAPE-42 offers a valuable instrument for investigating PEs in the Hebrew-speaking population and facilitates cross-cultural studies.

## 1 Introduction

Psychotic experiences (PEs) affect approximately 7.2% of the general population, manifesting along a continuum from subclinical levels to severe psychotic disorders, such as schizophrenia (1–3). These experiences, even when not fully meeting diagnostic criteria for psychotic disorders, are significant risk factors that can impact normative behavior and frequently co-occur with other clinical conditions like affective and anxiety, and autism spectrum disorders (4–7). Despite this, the precise effects of PEs on typical behavior remain poorly understood (8). To advance our understanding of psychosis etiology, it is essential to use tools that measure the intensity and frequency of PEs in both clinical and non-clinical populations, and that capture individual differences in subdomains of PEs. Such tools can help differentiate between typical and pathological behavior, facilitating systematic modeling of psychosis-related conditions (1, 9), and providing a structured framework for directly investigating the interplay between psychotic disorders and other clinical conditions. The key example illustrating the latest need is the ongoing debate on comorbidity between autism and psychosis. While these disorders are defined as strictly distinct (10), some proposed an overlapping modeling of the two spectra, based on genetic, etiologic, and symptomatologic evidence (11–14). In this model, a co-occurrence of the two conditions predicts an aggravation of deficits. Alternatively, the diametric model suggests that the disorders lie at the opposite ends of a unidimensional spectrum, with typical functioning at the center (15, 16). This approach, supported mainly by genetic evidence (17, 18), suggests an opposite symptomatology and therefore, no possible co-occurrence. The revised diametric model allows for co-occurrence and assumes both overlapping and diametric symptomatology (19). Nevertheless, the effect of the relative combination of psychotic and autistic traits remains unclear. Similarly, their combined effects on conditions with high comorbidity (e.g., depression) are still discussed (7, 20–22). Understanding these complex interplays will provide insight into the variability in psychotic symptomatology.

Cross-cultural research is also crucial for understanding variations in the expression of PEs to adapt research and clinical tools to the targeted populations. Studies show that cultural and environmental differences can influence both the prevalence and the nature of PEs (23–25). Yet, these cultural aspects are still not well understood (26–28). This is perhaps because most tools were developed for the Western world, and often applied in non-Western cultures without validation, leading to over- or under-estimation of measurement. Therefore, to develop tools that are culturally appropriate and standardized for use in diverse populations, there is a need to adapt the instruments to other cultures and languages.

The Community Assessment of Psychic Experience (CAPE) is a widely used tool designed to assess PEs in non-clinical populations (29). This self-report questionnaire includes 42 items measuring the frequency of PEs and the associated distress. Unlike other scales such as the Prodromal Questionnaire – Brief version (i.e., self-report tool evaluating early signs of psychosis focused on the positive dimension (30)) and the Schizotypical Personality Questionnaire (i.e., self-report evaluating psychotic traits including positive symptoms, interpersonal deficits and disorganization (31)), the CAPE-42 measures a wider range of psychotic experiences, including positive (e.g., bizarre experiences, delusional ideation), negative (e.g., social withdrawal, and avolition) and depressive dimensions. This approach enables a more detailed understanding of the broad spectrum of PEs (32), making the CAPE-42 more suitable for broader epidemiological research in the general population and cross-cultural research (27, 28, 33). The specific composition of the questionnaire demonstrates good reliability and validity (32, 34), and shows strong predictive capabilities for anxiety, depression, and stress sensitivity (35). Furthermore, the scores in the dimensions evaluated by this self-questionnaire closely align with the scores from interview-based assessments (35). Therefore, the CAPE-42 has become one of the most commonly used tools to evaluate PEs in research, leading to a need for cross-cultural adaptation.

Despite extensive validation and application of the CAPE-42 across cultures and languages (e.g., English, French, Spanish, Persian, Italian (36–41)), there is a notable gap in its application within Semitic-speaking populations., generating a need to adapt this tool to Middle Eastern cultures and languages, like performed by Fekih-Romdhane et al. (33) with Arabic speakers in Lebanon, and in the present study with Hebrew speakers in Israel. Furthermore, we observe that cross-validation was not systematically performed in previous translations. This method ensures that the translated version keeps the psychometric properties of the original questionnaire, which is essential for the interpretability of findings from cross-cultural studies using different versions of the same tool.

To address this gap, in this study, we conducted translation and cross-validation of the CAPE-42 to Hebrew. Our aims were to (1) validate the CAPE-42 in the Hebrew language, (2) assess the validity of the new tool using various convergence analyses, and (3) demonstrate how the questionnaire can be used to assess co-occurence and interaction between PEs and other clinical spectra. To these ends, we performed translation and back-translation of the CAPE-42. Following the translation, we used an online version of the questionnaire to collect a large sample of the Hebrew-speaking population in Israel. Cross-validation analysis showed an overall very good internal consistency of the Hebrew version. Additionally, there was a strong association between the dimensions of the CAPE-42 and other scales measuring similar constructs. Finally, we demonstrated the suitability of the CAPE-42 to investigate the association between autistic and psychotic traits with depressive symptoms.

## 2 Methods

### 2.1 Participants

In total, 359 Israeli participated in the study. The mean age of the remaining sample (N= 349) was *m* = 28.6 years, *sd* = 8.19, and 75% were females. Demographic characteristics are displayed in **Table 1**. To participate in this study, participants were required to hold Israeli citizenship, reside in Israel, and be native Hebrew speakers. Using the Google Form restrictions, we ensured that subjects participated only once in the study. They all received monetary compensation, either as a 20 shekels gift card or in cash, and digital consent was obtained from all participants. Based on ethnicity questions, we excluded 10 participants who were unlikely to be native Hebrew speakers. The study protocol was approved by an Ethics Committee of the University of Haifa (046/20) according to the latest version of the Declaration of Helsinki (42).

**Table 1:** The table displays the socioeconomic characteristics of the participants.

Variables	N (%)
Age (mean ± sd)	28.6 ± 8.17
Gender	
Male	88 (25%)
Female	261 (75%)
Status	
Activity (worker, military service, national service)	91 (26%)
No activity (maternity leave, not able to work, retired, unemployed)	26 (7%)
Student	232 (67%)
Education	
Secondary or less	67 (19%)
University	282 (81%)
Age (mean ± sd)	28.5 ± 8.09

### 2.2 Measurements

All demographic questions and clinical questionnaires were presented and answered in Hebrew.

#### 2.2.1 Demographic questionnaire

The first part of the survey consisted of demographic questions evaluating various aspects of the participants’ lives. Categories included age, sex/gender (participants reported the same information for both gender and sex), ethnicity, religiosity, education, professional activity, socio-economical background, personal and family history for neurodevelopmental conditions and physical disorders, physical activities, hobbies, and interests.

#### 2.2.2 Community Assessment of Psychic Experiences (CAPE-42)

The CAPE-42 is a questionnaire consisting of 42 items designed to assess a spectrum of psychotic experiences, encompassing positive, negative, and depressive dimensions. The positive dimension evaluates symptoms adding to the participant’s experiences (e.g., delusions) through 20 items (see **Supplementary Table 1** for item details). The negative dimension evaluates symptoms characterized by the loss of typical behavior (e.g., avolition) through 14 items. Finally, the depressive dimension is assessed through eight items. Participants rate the frequency of their experiences on a scale from 1 (never) to 4 (nearly always), which sums to a total score ranging from 42 to 168. We used the translation/back-translation method to adapt the questionnaire into Hebrew. Initially, a native Hebrew speaker proficient in English translated the questionnaire from English to Hebrew. Subsequently, a native English speaker proficient in Hebrew performed the back-translation from Hebrew to English. Discrepancies between the two English versions were addressed by four researchers, comprising both native Hebrew and English speakers, and adjustments were made to the Hebrew version accordingly. The distribution of responses to each item is provided in **Supplementary Table 2**, and the Hebrew version of the CAPE-42 is provided in the **Supplementary Material, Appendix A**.

#### 2.2.3 Autism Spectrum Quotient (AQ-50)

The AQ questionnaire is a self-report tool designed to measure autistic traits in both general and clinical populations (43). It comprises 50 items organized into five dimensions (of 10 items each) of the autistic spectrum: social skills, attention to detail, attention switch, communication, and imagination. Each item is a statement, that participants respond to by indicating their level of agreement on a 4-point scale ranging from “definitely disagree” to “definitely agree”. Each item is scored as 0 or 1 based on agree/disagree, resulting in a total autistic quotient ranging from 0 to 50.

#### 2.2.4 Center for Epidemiologic Studies (CES-D)

The CES-D is a widely used self-report questionnaire designed to assess depressive symptoms in the general population (44), and is commonly used in research and clinical settings to screen for depressive symptoms and monitor changes in depression severity over time. It consists of 20 items covering various aspects of depression, including sadness, feelings of guilt, or sleep disturbance. Participants rate the frequency of their experiences over the past week on a 4-point scale ranging from “rarely or none of the time” to “most or all the time”. The items are scored from 0 to 4 and summed to obtain a total score, which ranges from 0 to 60.

#### 2.2.5 Prodromal Questionnaire — Brief Version (PQ-B)

The PQ-B questionnaire is a brief version of the 92-item Prodromal Questionnaire, a screening tool developed to evaluate symptoms indicative of the prodromal phase of psychosis, intending to identify individuals at high risk of developing psychotic disorders such as schizophrenia (45, 46). This short version retains 21 items assessing positive symptoms commonly observed in the prodromal phase. Participants respond to questions regarding personal experiences, indicating whether they have experienced each symptom and, if so, the extent to which these experiences have caused distress or impairment. Responses are scored on a 5-point Likert scale ranging from “strongly disagree” to “strongly agree”. Items answered “no” receive a score of 0, while those answered “yes” are scored from 1 to 5 based on the perceived impact of the experience. The total score is calculated as the sum of scores for all items, resulting in a score ranging from 0 to 105.

### 2.3 Procedures

The data were collected using a Google Form link between June and October 2023. After obtaining the digital consent, participants were informed about the study’s purpose (i.e., “to learn about certain aspects and experiences in your life”). They were assured of the questionnaire’s anonymity and encouraged to respond spontaneously without overthinking. The survey comprised 28 demographic questions, 50 questions from the AQ, 42 from the CAPE-42, 20 from the CES-D, and 21 from the PQ-B. An attentional check question was presented for every 30 items, and all participants succeeded in this task. Overall, participants answered 166 questions, which took approximately 30 minutes.

### 2.4 Data analyses

All analyses were executed in Rstudio version 4.3.2.

#### 2.4.1 Preliminary analyses

We calculated the means and standard deviations of all assessed scales and the subscales of the CAPE-42 for the overall sample and by gender.

#### 2.4.2 Confirmatory Factor Analysis (CFA)

The CFA was employed to evaluate the underlying structure of the variables set by examining the relationship between items and pre-defined latent factors. Employing the maximum likelihood method, we computed three parameters estimating the discrepancy between the observed covariance matrix and the model-implied covariance matrix. First, normed model chi-square ( $\frac{\chi 2}{df}$ ) values below 3 indicated a good fit, between 3 and 5 an acceptable fit, and above 5 a mediocre fit. Second, Steiger-Linod Root Mean Square Error of Approximation (RMSEA) values below 0.06 indicate a good fit, and between 0.08 and 0.1 an acceptable fit. Third, Standardized Root Mean Square Residual (SRMR) values below 0.08 indicated a good fit and between 0.08 and 0.1 an acceptable fit. Additionally, CFA enabled the estimation of parameters by comparing the fit of the proposed model to a null or baseline model where variables were uncorrelated, namely the Tucker-Lewis index (TLI) and the Comparative Fit Index (CFI). For both estimates, values above 0.90 showed a good fit, and values between 0.90 and 0.85 showed an acceptable fit (47).

We conducted a CFA on the eight-factor (first-order) model on the CAPE (i.e., depressive, social withdrawal, affective flattening, avolition, bizarre experiences, perceptual abnormalities, persecutory ideation, and magical thinking (33)), and a CFA of the three original factors that were added to the model as second-order factors (29). In this last model, the factor negative dimension included social withdrawal, affective flattening, and avolition, and the positive dimension included bizarre experiences, perceptual abnormalities, persecutory ideation, and magical thinking.

#### 2.4.3 Reliability

We used two main coefficients, the Cronbach’s α and McDonald’s ω, to assess the composite reliability of the translated version of the CAPE-42. Cronbach’s α is widely used to measure the internal consistency, by assessing the degree to which items within the instrument are interrelated (48, 49). However, because this coefficient may be influenced by both common and unique variances among items, which can lead to overestimated or underestimated reliability (50, 51), we also calculated omega (52), a more accurate assessment of reliability in tools involving multidimensionality as in the CAPE-42 (53, 54). A commonly accepted threshold for satisfactory reliability is 0.70 or higher. The omega and alpha coefficients indicated a very good reliability for the AQ (*ω* = 0.79, *α* = 0.81), CES-D (*ω* = 0.95, *α* = 0.93), and PQ-B (*ω* = 0.90, *α* = 0.88).

#### 2.4.4 Convergence and predictive score

To evaluate the convergent validity of the translated version of the CAPE-42 in Hebrew, we estimated the convergence between subscales and other scores supposedly evaluating the same constructs. Here, the convergence was evaluated between the positive symptoms scores with the score derived from the PQ-B, and the depressive score with the score derived from the CES-D, using Pearson’s correlation. In addition, we evaluated the predictive validity of the translated CAPE-42 by calculating the Pearson’s correlation coefficient with the AQ and CES-D. The objective was to identify correlations that align with existing literature, providing a benchmark for expected associations between specific subscales of the CAPE-42 and corresponding dimensions of the AQ and CES-D (7). Correlations with values less than 0.10 were categorized as very weak, between 0.10 and 0.30 as weak, between 0.30 and 0.50 as moderate, between 0.50 and 0.70 as strong, and above 0.70 as very strong (55).

#### 2.4.5 Gender invariance

Finally, to ensure that the translated version of the CAPE-42 was free from gender bias, we rigorously examined gender invariance as part of the validation process. By conducting independent t-tests, we aimed to identify potential gender-related variations in the scores.

#### 2.4.6 Predictive model

There is a complex interplay between psychotic experiences and autistic traits in relation to depressive symptoms (7, 22). Previous studies mainly focused on the combined effect of autistic and positive psychotic traits. However, the revised diametric model suggests that the co-occurrence between autism and psychosis may manifest by an overlap between autistic and negative psychotic traits, highlighting the importance of including the negative psychotic subscale. Therefore, we aimed to elucidate how autistic, positive psychotic, and negative psychotic traits individually and synergistically influence depressive symptomatology by conducting a linear regression analysis that controlled for the potential confounding effect of *Gender* using the following model:

CES-D ∼ AQ + CAPEpos + CAPEpos × AQ + CAPEneg + CAPEneg × AQ + Gender.

#### 2.4.7 Principal component analysis

We conducted a Principal Component Analysis (PCA) to identify the latent structure between the AQ and the CAPE-42. Here, we aimed to compare the extracted structure with previous findings that conducted similar analyses on the two scales (19, 59, 60) to assess whether the component pattern observed in our study aligns with those identified in other versions of the questionnaire. Furthermore, we aimed to use the extracted structure to test the diametrical model, a dominant view suggesting an opposition between positive psychotic and autistic traits (15, 16, 56, 57), and a co-occurrence between negative psychotic and autistic traits (19, 58). We conducted a PCA on the five subscales of the AQ and 7 subscales of the CAPE-42. The depressive subscale of the CAPE-42 was excluded from the analyses to compare the results with studies that performed a PCA between autistic and psychotic scales (19, 59, 60). To determine the principal components to retain we used the eigenvalue > 1 criterion. The analysis was performed using a correlation matrix rather than the covariance matrix to standardize the variance of the variables and we did not apply any rotations to the components, similar to Nenadić et al. (19). To adjust for potential sample error-induced inflation of eigenvalues, we conducted Horn’s parallel analysis. Here, we expected a common latent structure between factors measuring negative psychotic and autistic traits, and an opposite latent structure between factors evaluating positive psychotic and autistic traits.

#### 2.4.8 Canonical correlation analysis

To further explore the nature of the association between the dimensions of psychotic and autistic traits, we performed Canonical Correlation Analysis (CCA) (61) on the subscales of the AQ and CAPE-42, excluding the depressive dimension of the CAPE-42. CCA is a multivariate technique that identifies linear combinations of variables (i.e., canonical variates, or CVs) from each set that maximize the shared variance between the two datasets. The strength of these relationships was assessed using the canonical correlation coefficient *r_c_* for each CV. Wilks’ lambda, a likelihood ratio test, was used to evaluate the statistical significance of the association between the canonical variates (62). In addition, we examined key parameters, including the eigenvalue, the cumulative explained variance, and the shared explained variance, indicating respectively the amount of variance explained by each CV, the variance accounted for by the most significant CVs, and the extent to which the variance in one set is explained by the variance in the other set. Canonical loadings and standardized coefficients were examined to interpret the magnitude and direction of the contributions of each variable (i.e., subscale) to the CV. We considered variables with canonical loadings greater than 0.3 (in absolute value), and greater than their contribution in other CV, to strongly contribute to the CV of interest (63). The analysis was performed with the R packages yacca and CCP.

## 3 Results

### 3.1 Preliminary analyses

Our sample scored on the CAPE-42 with a total mean of *m* = 72.36 (*sd* = 15.66), and with means of *m* = 30.11 (*sd* = 6.91), *m* = 26.71 (*sd* = 6.83), *m* = 15.55 (*sd* = 4.60) on the positive, negative, and depressive subscales, respectively. Notably, six participants (1.72%) scored above 50 on the positive subscale, surpassing this suggested cut-off value (64). The summary of scores for all measured scales is provided in **Table 2**.

**Table 2:** Table summarizing the means and standard deviations for all assessed scales (CAPE-42, AQ, CES-D, PQ-B), and the subscales of the CAPE-42, presented for the overall sample and stratified by gender.

	Overall	Male	Female
Sample size	349	88	261
Age	28.6 ± 8.17	28.4 ± 6.71	28.7 ± 8.61
CAPE	72.36 ± 15.66	72.41 ± 16.09	72.34 ± 15.40
Positive	30.11 ± 6.91	31.78 ± 7.96	29.54 ± 6.43
Negative	26.71 ± 6.83	26.30 ± 6.87	26.85 ± 6.82
Depressive	15.55 ± 4.60	14.33 ± 4.04	15.95 ± 4.71
AQ	18.60 ± 6.37	19.19 ± 6.45	18.40 ± 6.34
CES-D	20.59 ± 12.79	18.47 ± 11.15	21.31 ± 13.24
PQ-B	13.80 ± 14.39	13.23 ± 13.71	14.00 ± 14.63

### 3.2 Confirmatory factor analysis

The CFA parameters of the eight-factor (first order) model of the CAPE indicated a fit from good (χ2/df = 1978.593/791 = 2.501, SRMR = 0.074, CFI = 0.804, and TLI = 0.787) to acceptable (RMSEA = 0.066 (90% CI 0.062 0.069)). The standardized estimates of factor loadings were adequate except for 6 items below 0.30 (**Supplementary Table 1**). The CFA fit of the eight-factor (first order) and the three-factor (second order) model showed a lesser good fit, with three parameters indicating a good fit (*χ2*/*df* = 2347.35/816 = 2.88, CFI = 0.748, TLI = 0.734, and two parameters indicating an acceptable fit (RMSEA = 0.073 (90% CI 0.070, 0.077), SRMR = 0.140). Furthermore, 24 items had factor loadings below the adequate threshold. With more parameters meeting the cutoff values indicating a good fit, and significantly more factor loadings being above the adequate threshold, we can conclude that the eight-factor model demonstrated the best fit for our data, consistent with the findings of Fekih‐Romdhane et al. (33) in their Arabic translation.

### 3.3 Reliability

The omega and alpha analyses revealed a general omega factor of *ω*_g =_ 0.94, and an alpha coefficient of *α* = 0.93, indicating a high overall internal consistency. We subsequently calculated the internal consistency of each factor, revealing robust reliability for seven of the eight factors: Depressive (*ω*_g_ = 0.90, *α* = 0.87), social withdrawal (*ω*_g_ = 0.56, *α* = 0.55), affective flattening (*ω*_g_ = 0.71, *α* = 0.70), avolition (*ω*_g_ = 0.86, *α* = 0.82), bizarre experiment (*ω*_g_ = 0.85, *α* = 0.75), perceptual abnormalities (*ω*_g_ = 0.79, *α* = 0.74), persecutory ideation (*ω*_g_ = 0.79, *α* = 0.71), and magical thinking (*ω*_g_ = 0.70, *α* = 0.63). To investigate the low reliability in the social withdrawal factor, we calculated the mean of inter-item correlation between the items included in the subscale. The mean inter-item correlation (*m_r_* = 0.29) was within the adequate range (0.15 < *m_r_* < 0.5, with *m_r_* < 0.15 indicating that the items do not measure the same construct, and *m_r_* > 0.50, indicating that the questions are too similar/redundant (65)). In the future, incorporating additional questions might be considered to improve the sensitivity of this dimension and to capture better the inter-individual variability. Overall, these results confirm that the Hebrew CAPE-42 has very good overall and subscale-level internal consistencies.

### 3.4 Convergence and predictive scores

Convergence of the CAPE-42 were evaluated using correlation analyses between the PQ-B and CES-D with the two subscales of the CAPE-42 measuring similar constructs. The correlation between the positive dimension of the CAPE-42 and the PQ-B, *r*(347) = 0.64, *p* <.001, and between the depressive dimension of the CAPE-42 and the CES-D score, *r*(347) = 0.81, *p* <.001, were very strong. The expected correlation between positive psychotic traits and autistic traits (7) was moderate, *r*(347) = 0.32, *p* <.001. The correlation matrix between the scores of the subscales of the CAPE-42 with the other measures is displayed in **Table 3**. These correlation coefficients show a close association between the dimensions and subscales of the CAPE-42 with scales evaluating similar constructs, demonstrating a robust convergent validity for the translated CAPE-42.

**Table 3:** Table of correlation coefficients (*r* values) between dimensions of the CAPE-42 with other total and dimensional measures.

Dimension	AQ total	AQ social	AQ detail	AQ switch	AQ com	AQ imag	CES-D	PQB
Positive	0.32***	0.17**	0.10	0.19*	0.37***	0.14*	0.47***	0.64***
Bizarre experiences	0.28***	0.14*	0.07	0.17**	0.30***	0.18**	0.39***	0.56***
Perceptual abnormalities	0.19*	0.09	-0.02	0.11	0.28***	0.16**	0.26***	0.47***
Persecutory ideation	0.38***	0.26***	0.10	0.29***	0.40***	0.11	0.60***	0.64***
Magical thinking	0.09	-0.01	0.11	-0.01	0.19**	0	0.12	0.33***
Negative	0.47***	0.50***	-0.03	0.42***	0.40***	0.14*	0.75***	0.55***
Social withdrawal	0.45***	0.49***	0.02	0.42***	0.33***	0.09	0.60***	0.37***
Affective flattening	0.30***	0.34***	-0.07	0.25***	0.30***	0.10	0.57***	0.48***
Avolition	0.46***	0.47***	-0.04	0.41***	0.38***	0.16*	0.73***	0.54***
Depressive	0.47***	0.42***	-0.02	0.40***	0.46***	0.14	0.81***	0.54***
Total	0.47***	0.42***	0.03	0.39***	0.45***	0.13*	0.77***	0.68***

p-values were adjusted using the False Discovery Rate method (q < 0.05).

*q < 0.05), **q < 0.01, ***q <0.001.

### 3.5 Gender invariance

The unpaired t-tests (**Table 4**) revealed no significant difference between males and females in the total score of the CAPE-42 after applying FDR corrections (*q* represents the corrected p-values), *t*(145.52) = 0.03, *q* = 0.974, the negative dimension, *t*(148.93) = 0.65, *q* = 0.708 and the positive dimension, *t*(127.45) = 2.39, *p* = 0.070. However, significant gender differences were observed in two subscales of the positive dimension, bizarre experiences, *t*(125.59) = 2.83, *q* = 0.020, and perceptual abnormalities, *t*(114.51) = 3.19, *q* = 0.011, where males showed significantly higher scores. Moreover, we found a difference in the depressive dimension, with females exhibiting a significantly higher score than males, *t*(173.06) = 3.13, *q* = .011, consistent with Fekih-Romdhane et al. (33). These results indicate that the Hebrew CAPE-42 does not generate gender biases and captures only the gender-based disparity in depressive traits within psychotic conditions (66).

**Table 4:** Table displaying results of t-tests analyzing the differences between gender in the dimensions and the total score of the CAPE-42.

Dimension	Males Mean ± sd	Females Mean ± sd	*t*	*f*	*p*	*q*
Positive	31.78 ± 7.96	29.54 ± 6.43	2.39	127.45	0.018	0.070
Bizarre experiences	8.93 ± 2.90	7.97 ± 2.29	2.83	125.59	0.005	0.020*
Perceptual abnormalities	3.61 ± 1.17	3.19 ± 0.78	3.19	114.51	0.002	0.011*
Persecutory ideation	11.65 ± 3.00	11.35 ± 2.89	0.82	145.17	0.416	0.654
Magical thinking	7.59 ± 2.52	7.03 ± 2.23	1.84	135.94	0.067	0.148
Negative	26.30 ± 6.87	26.85 ± 6.82	-0.65	148.93	0.515	0.708
Social withdrawal	6.11 ± 1.75	6.03 ± 1.81	0.38	154.41	0.704	0.774
Affective flattening	5.03 ± 1.80	4.92 ± 1.81	0.50	150.70	0.618	0.755
Avolition	15.15 ± 4.03	15.89 ± 4.10	-1.49	152.06	0.138	0.253
Depressive	14.33 ± 4.04	15.95 ± 4.71	-3.13	173.06	0.002	0.011*
Total	72.41 ± 16.09	72.34 ± 15.40	0.03	145.52	0.974	0.974

q-values represent p-values corrected for multiple comparisons using the False Discovery Rate (FDR) method.

Significance levels: *q < 0.05.

### 3.6 Predictive model

A linear regression analysis was conducted to investigate the independent effects of AQ, CAPE positive, CAPE negative, and the interaction between AQ and CAPE positive and between AQ and CAPE negative on the CES-D score, while controlling for Gender. The regression model was statistically significant and explained approximately 59.30% of the variance of the CES-D score (*F*(6, 342) = 83.04, *p* <.001, adjusted *R²* = .586). The results indicate that the AQ score (*B* = 2.12, *se* = 0.51, *t* = 4.17, *p* <.001), the CAPE positive score (*B* = 1.18, *se* = 0.59, *t* = 1.98, *p* = .049), and the CAPE negative score (*B* = 8.15, *se* = 0.60, *t* = 13.68, *p* <.001) were significant positive predictors of the CES-D score. The variable Gender (male) was a significant negative predictor (*B* = -2.85, *se* = 1.04, *t* = -2.74, *p* = .007). The interaction between AQ and CAPE positive scores (*B* = 0.34, *se* = 0.59, *t* = 0.59, *p* = .558), as well as the interaction between AQ and CAPE negative scores (*B* = -0.89, *se* = 0.51, *t* = -1.76, *p* = .079) were not significant. These findings suggest that autistic, positive and negative psychotic traits independently contribute to depressive symptoms. Furthermore, the results are consistent with the gender invariance analysis, confirming that participants’ gender is a predictor of depressive symptoms.

### 3.7 Principal component analysis

**Figure 1:**
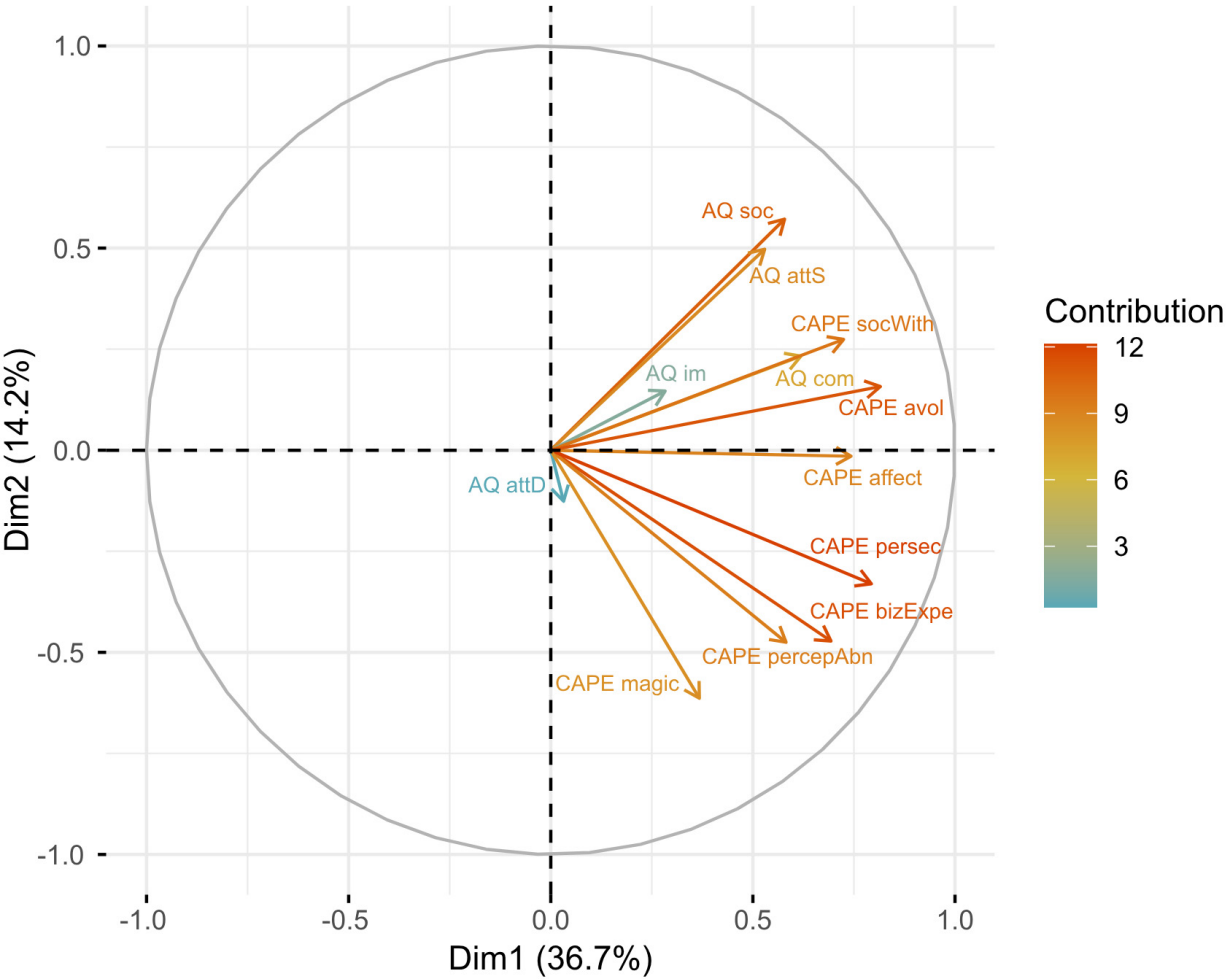
Visualization of the PCA illustrating the contribution of seven factors from the CAPE-42 and five factors from the AQ to the two principal components extracted. The position along the axes indicates the extent to which a variable aligns with the patterns of variation captured by the first (x-axis) and the second (y-axis) components. The results indicate that most AQ subscales and two subscales of the CAPE-42 measuring negative symptoms (i.e., avolition and social withdrawal) have opposite loadings (i.e., positive values in Dimension 2) to the positive subscales of the CAPE-42 and the attention to detail subscale of the AQ (i.e., negative values in Dimension 2). The affective flattening from the negative dimension of the CAPE-42 effectively has no contribution to the second factor (value = 0.01). This demonstrates a diametric structure between dimensions evaluating negative psychotic and autistic traits, on the one hand, and positive psychotic experiences on the other.

The PCA of the AQ and the CAPE-42 subscales identified four principal components. Two principal components were retained after correcting for sample error-induced inflation of eigenvalues, using Horn’s parallel analysis (see **Figure 1** for factor loadings in the two retained principal components, and **Supplementary Table 3** for the factor loadings in the four original principal components). The first principal component explained 36.69% of the variance (*m* = 36.69, *sd* = 2.10) and the second accounted for 14.17% (*m* = 14.17, *sd* = 1.70), together, totaling 50.86% of the variance. All factors positively loaded on the first component. However, we observed a diametric structure in the second component between subscales measuring negative psychotic and autistic traits, and positive psychotic traits. Factors related to autistic traits (i.e., social skills, attention switch, communication, imagination) and negative psychotic traits (i.e., avolition and social withdrawal) showed positive loadings, while factors associated with positive psychotic traits (i.e., persecutory ideation, magical thinking, bizarre experience, perceptual abnormality), and attention to detail showed negative loadings (see **Table 5**).

**Table 5:** Factor loadings from the PCA for the subscales of the CAPE-42 and the AQ.

Scale	Dimension	Component	
		1	2
	Explained variance	36.69%	14.17%
AQ	Social skills	0.28	0.44
	Attention switch	0.25	0.38
	Attention detail	0.02	-0.10
	Communication	0.29	0.18
	Imagination	0.13	0.11
CAPE	Social withdrawal	0.35	0.21
	Affective flattening	0.35	0.01
	Avolition	0.39	0.12
	Bizarre experience	0.33	-0.36
	Perceptual abnormalities	0.28	-0.36
	Persecutory ideation	0.38	-0.25
	Magical thinking	0.18	-0.47

The shading highlights negative factor loadings (i.e., eigenvalue < 1) in the second component, indicating that subscales evaluating positive psychotic traits are inversely loaded compared to those evaluating negative psychotic and autistic traits.

The associations between the AQ and CAPE-42 subscales identified through the CCA closely reflected the component structure revealed by the PCA. Therefore, the results of the CCA are presented in Supplementary results and **Supplementary Tables 4**, **5**.

## 4 Discussion

The primary aim of this study was to examine the psychometric properties of the Hebrew translation of the CAPE-42 in a sample of Hebrew-speaking adults from the general population in Israel.

We systematically translated and validated the CAPE-42 using the translation/back-translation method, and its association with the AQ, CES-D, and PQ-B. Overall, participants’ scores were consistent with those reported in the literature (35, 37, 38, 67), and only six participants (1.72%) exceeded the suggested cut-off value. This suggests that our sample included a small proportion of participants with elevated psychotic traits, indicating a higher risk for psychotic-related disorders. Alpha and omega reliability coefficients supported high internal consistency within the overall questionnaire and all factors, except social withdrawal. However, since reliability measures are not fully suitable for inter-item correlations with less than 10 items, the mean inter-item correlation of the social withdrawal subscale demonstrated good internal consistency despite the low reliability scores. The CFA indicated that the eight-factor model (33) fits our data better than the original structure proposed by Stefanis et al. (29), which adds the three original dimensions as a second-order factor. These findings suggest that the three dimensions might obscure the variability within and between individual experiences when modeling PEs. In contrast, the eight-factor model provides a more nuanced representation of the internal structure, reflecting better the multifactorial nature of psychosis and PEs. Therefore, we recommend using the subscale scores for research and deep phenotyping.

Furthermore, gender analysis showed no evidence for a gender-biased translation, with gender differences only appearing in dimensions consistent with previous findings, such as the depressive dimension. Finally, we conducted a predictive model to evaluate the contribution of autistic (i.e., AQ) and psychotic (i.e., CAPE positive and negative) traits to depressive scores (i.e., CES-D), while controlling for gender. The results showed that autistic and psychotic traits independently predict higher depression scores, while the male gender was a negative predictor.

We found strong correlations between dimensions of the CAPE-42 and other scales measuring similar constructs, supporting the validation of the new tool. We also found a moderate correlation between the positive dimension of the CAPE-42 and autistic traits. This moderate correlation contributes to the current ongoing debate regarding the association between autistic and psychotic traits. Previous research has produced mixed results; some studies found no correlation between these traits (68, 69), others reported a negative correlation (56, 58), and some a positive correlation (70, 71). Additionally, the PCA results, indicating an opposite loading of factors measuring autistic and negative psychotic traits, and positive psychotic traits, are aligned with previous research investigating the structure of tools evaluating autistic and psychotic traits (19, 59). These findings support the diametric model (15, 16, 56, 57), which places autism and psychosis at opposite ends of a single spectrum, as well as the revised diametrical model (19, 58), which suggests that while negative psychotic and autistic traits overlap, positive psychotic traits oppose autistic traits.

Moreover, these results indicate that investigating the overlap between the two conditions provides a valuable framework for understanding their comorbidity. Evidence indicates that individuals with autism have an increased risk of developing psychosis, and vice versa (72, 73). While these findings are generally attributed to shared genetic (74–76) and neurobiological factors (77, 78), the symptom overlap between the two conditions may also play a role (79). However, distinguishing overlapping symptoms from clinical comorbidity remains challenging due to the limitations in current diagnostic tools, potentially leading to mis- or underdiagnosis. Indeed, we believe that measuring autistic traits/symptoms in psychosis, and vice versa, requires tools specifically designed for the clinical populations, by considering the overlapping symptoms, as well as possible differences in the expression of symptoms due to the co-occurrence (80, 81).

This study provides a significant and reliable tool for research and clinical purposes in assessing PEs and related disorders within the Hebrew-speaking population. In the future, this adapted questionnaire will allow for the screening of psychotic experiences in both non-clinical and clinical populations, which in turn will aid in the understanding of the deep phenotyping of psychotic disorders, and identifying traits that may serve as early indicators of clinical conditions. Additionally, this tool will help to explore the implication of PEs in other clinical conditions, either as protective, aggravating factors, or comorbidity. For example, our research provides insight into the independent effect of autistic and psychotic traits on depressive symptoms (7, 22). While comorbidity in clinical conditions typically exacerbates depressive symptoms (82), our findings suggest that there is no enhancement of depressive symptoms from the interaction between autistic and positive psychotic traits. Nonetheless, this contributes to the growing body of research supporting the need to examine the interaction between trait dimensions in order to better understand their combined impact on outcomes (7, 71, 83).

Finally, this research provides a valuable tool that can be integrated into cross-cultural studies, crucial for understanding variations in PEs across different cultures and the implication of the cultural factor in symptom modulation. Studies have shown a higher frequency of PEs among minority groups living in Western countries (23–25, 84). Identifying the risk factors that contribute to differences in the prevalence of psychotic disorders is essential for adapting diagnosis tools (85) and improving patient rehabilitations, though their investigation represents significant challenges. Differences in prevalence may be influenced by genetic, cultural, or social-environmental factors. Previous research that investigated population differences based on countries’ socioeconomic status yielded inconsistent results. For example, some studies found a higher frequency of PEs is middle/high-income countries (26), while others found higher frequencies in low/middle-income countries, with greater distress level reported in high-income countries (27). Another challenge in cross-cultural studies is the variation in the expressions of the PEs, which can be influenced by religious beliefs, help-seeking behavior, and societal stigmatization (86). Therefore, the CAPE-42 in Hebrew will enable the inclusion of the Hebrew-speaking population in cross-cultural studies, a relevant population to disentangle some genetic, cultural, and social-environmental risk factors due to some unique characteristics of the Hebrew-speaking population in Israel (e.g., high-income country, local and global geopolitical conflict, Semitic culture, inter-generational history, and diverse genetic origins [e.g., European Middle-Eastern, North-African, Ethiopian]).

### 4.1 Study limitations

Here, we validated the CAPE-42 in Hebrew over a restricted population (i.e., primarily students, and 75% of females). As such, the sample may not fully represent the general population, which could limit the generalization of our findings. However, it is important to highlight that the demographic items revealed significant diversity within our sample (e.g., salary, religiosity, marital situation, and geographic area of residence).

Furthermore, the sample is only from a non-clinical population. However, a screening tool must distinguish between clinical and non-clinical individuals, and accurately assess psychotic experiences within the clinical population. This is particularly challenging for conditions like psychotic disorders where the main symptoms, such as delusions, can affect self-awareness. Therefore, the tool must be validated on both groups to evaluate additional psychometric properties, such as the sensitivity, specificity, and cutoff value. To our knowledge, only Boonstra et al. (64) measured these psychometric characteristics, proposing a cutoff of 50 on the positive dimension, which results in a sensitivity of 77% and specificity of 71%. While our findings show that the tool demonstrated good reliability in the non-clinical population, its validity within the clinical population should be tested in future research. Moreover, the tool’s validation could have benefited from gold-standard clinical interviews to control for potential biases associated with self-report tools (e.g., social desirability, variability in the understanding of the questions).

## 5 Conclusions

The properties of the CAPE-42 make it a crucial tool for evaluating PEs in both research and clinical environments. This study, following a rigorous translation and validation procedure, provides a validated version of the questionnaire, with high psychometric qualities, for the Hebrew-speaking population. This tool is essential for screening PEs in both clinical and non-clinical populations in the perspective of a better understanding of the phenotype and early signs of psychotic disorders, and the comorbidity and interactions with other conditions. Furthermore, it could be employed to investigate cross-cultural differences in the expression of PEs, a recent lead that could provide insights into the implications of culture and environment on the manifestations of PEs.

## Data Availability

The datasets presented in this study can be found in online repositories. The names of the repository/repositories and accession number(s) can be found below: https://osf.io/kwjyr/?view_only=a2a1272052c64ed0b1da262f0bacef06.
