# Supplementary material for: Validation of the Hebrew version of the Community Assessment of Psychic Experiences in a sample of Israeli Hebrew speakers
Source: Front Psychiatry. 2025 May 14;16:1548310. doi: 10.3389/fpsyt.2025.1548310 (PMC12116564; doi:10.3389/fpsyt.2025.1548310)
Supplement: Supplementary file 1 [file Table1.docx]

**Supplementary material**

**Supplementary results.**

**Canonical Correlation Analysis.**

The CCA was conducted between the five subscales of the AQ (*social skills, attention to detail, attention switching, communication, and imagination*) and seven subscales of the CAPE-42 (*bizarre experiences, perceptual abnormalities, persecutory ideation, magical thinking, social withdrawal, affective flattening, and avolition*) to examine the relationship between the autistic and psychotic subdimensions. A total of five canonical variates were extracted from the analysis. However, only the first two canonical variates (CV 1 and CV 2) exhibited canonical correlations greater than .30, indicating moderate to strong correlation, and reached statistical significance (*p* < .05). Specifically, the values were *r_c_* = .59 (eigenvalue = .53) for CV 1, and *r_c_* = .37 (eigenvalue = .16) for CV 2. Furthermore, CV 1 accounted for 34.51% of the variance shared between the two sets, and CV 2 accounted for 13.50%. The cumulative proportion of explained variance reached 88.03% (**Supplementary table 4**). Therefore, these two canonical variables were retained for the canonical loading analysis (**Supplementary table 5**).

We extracted the standardized canonical correlation coefficients to quantify the contribution of each variable (i.e., subscale) to CV 1 and CV 2. For each subscale, the absolute value of the coefficient reflects the magnitude of the contribution to the CV, while the sign indicates the direction of the contribution. The results revealed that the negative subscales of the CAPE-42 (i.e., social withdrawal, affective flattening, avolition) and three of the AQ subscales (i.e., social skills, attention switching, communication) negatively contributed to CV 1. In contrast, all positive subscales of the CAPE-42 (i.e., bizarre experience, perceptual abnormalities, persecutory ideation, magical thinking), and two AQ subscales (i.e., attention to detail, communication), positively contributed to CV 2.

These findings highlight two distinct patterns of association between autistic and psychotic traits. The results suggest a positive relationship between psychotic and autistic traits, driven by the negative psychotic dimension, while positive psychotic traits show minimum association with autistic traits. This distinction suggests that while social difficulties are a shared feature across autism and psychosis-proneness (CV1), cognitive-perceptual atypicalities are more closely tied to psychotic-like traits (CV2) but may also interact with specific autistic traits, namely communication difficulties and attention to detail. Communication difficulties in CV2 suggest that atypical perceptual and cognitive experiences may impair social information processing, aligning with findings that psychotic-like traits often involve disorganized thinking. Additionally, attention to detail may reflect an intersection between autism-related perceptual focus and psychosis-proneness, potentially leading to rigid or idiosyncratic interpretations of sensory and social information, further affecting communication. Overall, these results are consistent with the PCA, and indicate a complex relationship that is both diametric and overlapping between the autistic and psychotic phenotypes.

**Supplementary tables**

**Supplementary table 1**. Table displaying standardized latent variable estimates

from the CFA of the eight-factor model and the CFA of the eight-factor model

with 3-factors as second-order factors.

| Item | Factor loading | |
| --- | --- | --- |
| Factor I/1: depressive dimension | 8-factor model | 8/3-factor model |
| 1 | 0.43 | 0.37 |
| 9 | 0.60 | 0.53 |
| 12 | 0.67 | 0.59 |
| 14 | 0.49 | 0.44 |
| 19 | 0.30 | 0.26 |
| 38 | 0.59 | 0.50 |
| 39 | 0.69 | 0.59 |
| 40 | 0.54 | 0.46 |
| Factor II: negative dimension |  |  |
| Factor 2: social withdrawal |  |  |
| 3 | 0.63 | 0.46 |
| 4 | 0.30 | 0.20 |
| 29 | 0.39 | 0.23 |
| Factor 3: affective flattening |  |  |
| 8 | 0.42 | 0.30 |
| 27 | 0.53 | 0.31 |
| 32 | 0.55 | 0.37 |
| Factor 4: avolition |  |  |
| 16 | 0.38 | 0.23 |
| 37 | 0.49 | 0.32 |
| 18 | 0.57 | 0.36 |
| 21 | 0.24 | 0.14 |
| 23 | 0.59 | 0.38 |
| 25 | 0.39 | 0.25 |
| 35 | 0.63 | 0.40 |
| 36 | 0.44 | 0.28 |
| Factor III: positive dimension |  |  |
| Factor 5: bizarre experiences |  |  |
| 17 | 0.21 | 0.13 |
| 24 | 0.37 | 0.24 |
| 26 | 0.32 | 0.21 |
| 28 | 0.43 | 0.28 |
| 30 | 0.46 | 0.30 |
| 31 | 0.63 | 0.23 |
| Factor 6: perceptual abnormalities |  |  |
| 33 | 0.31 | 0.21 |
| 34 | 0.32 | 0.21 |
| 42 | 0.18 | 0.12 |
| Factor 7: persecutory ideation |  |  |
| 2 | 0.37 | 0.26 |
| 5 | 0.32 | 0.23 |
| 6 | 0.24 | 0.16 |
| 7 | 0.38 | 0.26 |
| 10 | 0.37 | 0.26 |
| 22 | 0.46 | 0.27 |
| 41 | 0.26 | 0.16 |
| Factor 8: magical thinking |  |  |
| 11 | 0.68 | 0.40 |
| 13 | 0.59 | 0.38 |
| 15 | 0.33 | 0.22 |
| 20 | 0.28 | 0.21 |

**Supplementary table 2.** Frequency of distribution (%) of sample Responses for the CAPE-42 questionnaire (in English).

| Items | Never | Sometimes | Often | Nearly always |
| --- | --- | --- | --- | --- |
| 1. Do you ever feel sad? | 3.06 | 65.74 | 21.17 | 7.24 |
| 2. Do you ever feel as if people seem to drop hints about you or say things with a double meaning? | 23.68 | 55.43 | 13.37 | 4.74 |
| 3. Do you ever feel that you are not a very animated person? | 39.83 | 42.06 | 8.91 | 6.41 |
| 4. Do you ever feel that you are not much of a talker when you are conversing with other people? | 17.83 | 53.2 | 18.11 | 8.08 |
| 5. Do you ever feel as if things in magazines or on TV were written especially for you? | 52.37 | 35.1 | 8.36 | 1.39 |
| 6. Do you ever feel as if some people are not what they seem to be? | 6.13 | 60.17 | 24.79 | 6.13 |
| 7. Do you ever feel as if you are being persecuted in some way? | 7..54 | 16.99 | 3.9 | 2.79 |
| 8. Do you ever feel that you experience few or no emotions at important events? | 37.33 | 46.24 | 10.31 | 3.34 |
| 9. Do you ever feel pessimistic about everything? | 33.7 | 45.4 | 11.42 | 6.69 |
| 10. Do you ever feel as if there is a conspiracy against you? | 74.37 | 17.27 | 3.06 | 2.51 |
| 11. Do you ever feel as if you are destined to be someone very important? | 32.31 | 37.6 | 15.32 | 11.98 |
| 12. Do you ever feel as if there is no future for you? | 52.92 | 32.03 | 7.24 | 5.01 |
| 13. Do you ever feel that you are a very special or unusual person? | 25.63 | 40.67 | 19.5 | 11.42 |
| 14. Do you ever feel as if you do not want to live anymore? | 67.13 | 22.28 | 4.46 | 3.34 |
| 15. Do you ever think that people can communicate telepathically? | 53.2 | 36.49 | 5.29 | 2.23 |
| 16. Do you ever feel that you have no interest to be with other people? | 18.38 | 61.28 | 13.65 | 3.9 |
| 17. Do you ever feel as if electrical devices such as computers can influence the way you think? | 33.98 | 35.93 | 19.5 | 7.8 |
| 18. Do you ever feel that you are lacking in motivation to do things? | 6.96 | 52.09 | 27.02 | 11.14 |
| 19. Do you ever cry about nothing? | 47.63 | 40.95 | 6.13 | 2.51 |
| 20. Do you believe in the power of witchcraft, voodoo or the occult? | 69.08 | 21.45 | 4.74 | 1.95 |
| 21. Do you ever feel that you are lacking in energy? | 5.85 | 54.32 | 25.35 | 11.7 |
| 22. Do you ever feel that people look at you oddly because of your appearance? | 44.29 | 37.6 | 10.58 | 4.74 |
| 23. Do you ever feel that your mind is empty? | 64.35 | 27.02 | 3.06 | 2.79 |
| 24. Do you ever feel as if the thoughts in your head are being taken away from you? | 82.73 | 10.58 | 1.95 | 1.95 |
| 25. Do you ever feel that you are spending all your days doing nothing? | 27.02 | 4903 | 15.04 | 6.13 |
| 26. Do you ever feel as if the thoughts in your head are not your own? | 84.68 | 8.64 | 2.79 | 1.11 |
| 27. Do you ever feel that your feelings are lacking in intensity? | 59.61 | 28.13 | 6.41 | 3.06 |
| 28. Have your thoughts ever been so vivid that you were worried other people would hear them? | 72.7 | 19.22 | 3.9 | 1.39 |
| 29. Do you ever feel that you are lacking in spontaneity? | 21.17 | 54.32 | 15.6 | 6.13 |
| 30. Do you ever hear your own thoughts being echoed back to you? | 76.6 | 15.32 | 3.34 | 1.95 |
| 31. Do you ever feel as if you are under the control of some force or power other than yourself? | 82.73 | 11.14 | 1.39 | 1.95 |
| 32. Do you ever feel that your emotions are blunted? | 48.47 | 37.6 | 8.36 | 2.79 |
| 33. Do you ever hear voices when you are alone? | 88.3 | 7.24 | 1.11 | 0.56 |
| 34. Do you ever hear voices talking to each other when you are alone? | 91.36 | 3.9 | 1.67 | 0.28 |
| 36. Do you ever feel that you can never get things done? | 38.16 | 45.13 | 8.64 | 5.29 |
| 37. Do you ever feel that you have only a few hobbies or interests? | 35.93 | 44.85 | 11.7 | 4.74 |
| 38. Do you ever feel guilty? | 21.73 | 49.58 | 17.27 | 8.64 |
| 39. Do you ever feel like a failure? | 29.53 | 48.47 | 11.7 | 7.52 |
| 40. Do you ever feel tense? | 5.01 | 49.03 | 30.92 | 12.26 |
| 41. Do you ever feel as if a double has taken the place of a family member, friend or acquaintance? | 88.3 | 6.69 | 0.84 | 1.39 |
| 42. Do you ever see objects, people or animals that other people cannot see? | 89.42 | 6.69 | 0.56 | 0.56 |

**Supplementary table 3.** PCA results from the original analysis containing four

principal components, before adjusting for sample error-induced inflation of

eigenvalues.

|  |  | Component | | | |
| --- | --- | --- | --- | --- | --- |
|  |  | 1 | 2 | 3 | 4 |
|  | Explained variance | 36.69% | 14.17% | 9.42% | 8.55% |
| AQ | Social skills | 0.28 | 0.44 | 0.15 | 0.10 |
|  | Attention switch | 0.25 | 0.38 | 0.13 | 0.06 |
|  | Attention detail | 0.02 | -0.10 | 0.53 | 0.75 |
|  | Communication | 0.29 | 0.18 | 0.30 | -0.24 |
|  | Imagination | 0.13 | 0.11 | 0.58 | -0.46 |
| CAPE | Social withdrawal | 0.35 | 0.21 | -0.25 | 0.21 |
|  | Affective flattening | 0.35 | -0.01 | -0.34 | 0.04 |
|  | Avolition | 0.39 | 0.12 | -0.24 | 0.08 |
|  | Bizarre experience | 0.33 | -0.36 | 0.05 | -0.08 |
|  | Perceptual abnormalities | 0.28 | -0.36 | 0.04 | -0.26 |
|  | Persecutory ideation | 0.38 | -0.25 | -0.01 | 0.10 |
|  | Magical thinking | 0.18 | -0.47 | 0.15 | 0.12 |

**Supplementary table 4.** Results of the Canonical Correlation Analyses testing the associations between AQ and CAPE-42 subscales. The statistical significance of the canonical correlation (*r_c_*) for each canonical variate (CV) was assessed using the likelihood ratio test Wilks’ Lambda.

|  | *r_c_* | Eigenvalue | Cumulative contribution % | Shared variance % | Wilks’ Lambda | *p*-value | Approximate *F* value |
| --- | --- | --- | --- | --- | --- | --- | --- |
| CV 1 | .59 | .53 | 67.90 | 35.51 | .52 | < .001 | 6.88 |
| CV 2 | .37 | .16 | 88.03 | 13.50 | .79 | < .001 | 3.44 |
| CV 3 | .24 | .06 | 96.14 | 5.92 | .91 | .009 | 2.08 |
| CV 4 | .16 | .03 | 99.40 | 2.47 | .98 | .256 | 1.27 |
| CV 5 | .07 | < .01 | 100 | < 1 | .99 | .664 | 0.53 |

**Supplementary table 5.** Standardized canonical coefficients

of each variable for the first two canonical variates.

|  | CV 1 | CV 2 |
| --- | --- | --- |
| CAPE |  |  |
| Social withdrawal | -.90 | -.02 |
| Affective flattening | -.63 | .06 |
| Avolition | -.90 | .11 |
| Bizarre experience | -.34 | .67 |
| Perceptual abnormalities | -.24 | .62 |
| Persecutory ideation | -.58 | .74 |
| Magical thinking | -.02 | .69 |
| AQ |  |  |
| Social skills | -.90 | -.18 |
| Attention switch | -.79 | .07 |
| Attention detail | .01 | .39 |
| Communication | -.64 | .65 |
| Imagination | -.21 | .24 |

**Appendix A**. The validated Hebrew-translated version of the Community Assessment of Psychic Experiences (CAPE-42) and scoring instructions.

| **בחר/י את התשובה המתאימה לך ביותר על ידי סימון התיבה המתאימה.** | | | | **הוראות** |  |
| --- | --- | --- | --- | --- | --- |
| **כמעט כל הזמן** | **לעיתים קרובות** | **לעיתים** | **לעולם לא** | **שאלות** |  |
|  |  |  |  | ?האם קורה שאתה מרגיש עצוב | 1. |
|  |  |  |  | האם קורה שאתה מרגיש שאנשים רומזים לגביך או אומרים דברים בעלי משמעות כפולה? | 2. |
|  |  |  |  | האם קורה שאתה מרגיש שאתה אדם חסר חיות? | 3. |
|  |  |  |  | האם קורה שאתה מרגיש שאתה לא מאוד דברן כאשר אתה משוחח עם אנשים אחרים? | 4. |
|  |  |  |  | האם קורה שאתה מרגיש שדברים בעיתון או בטלוויזיה נכתבו במיוחד עבורך? | 5. |
|  |  |  |  | האם קורה שאתה מרגיש שאנשים מסוימים הם לא מה שהם נראים? | 6. |
|  |  |  |  | האם קורה שאתה מרגיש נרדף בדרך כלשהי? | 7. |
|  |  |  |  | האם קורה שאת/ה מרגיש/ה שאינך חווה או חווה מעט רגשות באירועים חשובים? | 8. |
|  |  |  |  | האם קורה שאתה מרגיש פסימי לגבי הכל? | 9. |
|  |  |  |  | האם קורה שאת/ה מרגיש/ה שישנה קנוניה נגדך? | 10. |
|  |  |  |  | האם קורה שאת/ה מרגיש/ה שנועדת להיות מישהו מאוד חשוב? | 11. |
|  |  |  |  | האם קורה שאת/ה מרגיש/ה שאין לך עתיד? | 12. |
|  |  |  |  | האם קורה שאתה מרגיש שאתה אדם מאוד מיוחד או בלתי רגיל? | 13. |
|  |  |  |  | האם קורה שאת/ה מרגיש/ה שאתה לא רוצה לחיות יותר? | 14. |
|  |  |  |  | האם את/ה חושב/ת לפעמים שאנשים יכולים לתקשר באופן טלפתי? | 15. |
|  |  |  |  | האם את/ה מרגיש/ה לפעמים שאין לך עניין להיות עם אנשים אחרים? | 16. |
|  |  |  |  | האם את/ה מרגיש/ה לפעמים שמכשירים אלקטרוניים, כמו לדוגמה מחשב, יכולים להשפיע על הדרך בה את/ה חושב/ת? | 17. |
|  |  |  |  | האם את/ה מרגיש/ה לפעמים שחסרה לך מוטיבציה על מנת לעשות דברים? | 18. |
|  |  |  |  | האם קורה שאת/ה בוכה ללא סיבה? | 19. |
|  |  |  |  | האם את/ה מאמינה/ מאמין בכישוף, וודו או כוחות נסתרים? | 20. |
|  |  |  |  | האם את/ה מרגיש/ה לפעמים שחסרה לך אנרגיה? | 21 |
|  |  |  |  | האם את/ה מרגיש/ה לפעמים שאנשים מסתכלים עליך בצורה מוזרה בגלל המראה שלך? | 22. |
|  |  |  |  | האם את/ה מרגיש/ה לפעמים שהראש שלך ריק ממחשבות? | 23. |
|  |  |  |  | האם את/ה מרגיש/ה לפעמים שהמחשבות בראש שלך נלקחות ממך? | 24. |
|  |  |  |  | האם את/ה מרגיש/ה לפעמים שכל הימים שלך חולפים מבלי שעשית דבר? | 25. |
| **כמעט כל הזמן** | **לעיתים קרובות** | **לעיתים** | **לעולם לא** |  |  |
|  |  |  |  | האם את/ה מרגיש/ה לפעמים שהמחשבות בראש שלך הם של מישהו אחר? | 26. |
|  |  |  |  | האם את/ה מרגיש/ה לפעמים שהתחושות שלך חסרות עוצמה? | 27. |
|  |  |  |  | האם קרה לך שהמחשבות שלך היו מוחשיות עד כדי כך שדאגת שאחרים יכולים לשמוע אותן? | 28 |
|  |  |  |  | האם את/ה מרגיש/ה לפעמים שחסרה לך ספונטניות? | 29. |
|  |  |  |  | האם קורה לך שאת/ה שומע/ת את המחשבות שלך חוזרות אליך כמו הד? | 30. |
|  |  |  |  | האם קורה לך שאת/ה מרגיש/ה שאת/ה תחת השפעתו של כוח או שליטה של מישהו אחר? | 31. |
|  |  |  |  | האם את/ה מרגיש/ה לפעמים שהרגשות שלך קהים? | 32. |
|  |  |  |  | האם קורה שאת/ה שומע/ת קולות כשאת/ה לבד? | 33. |
|  |  |  |  | האם קורה שאת/ה שומע/ת קולות משוחחים ביניהם כשאת/ה לבד? | 34. |
|  |  |  |  | האם את/ה מרגיש/ה לפעמים שאת/ה מזניח/ה את ההופעה החיצונית או ההיגיינה שלך? | 35. |
|  |  |  |  | האם את/ה מרגיש/ה לפעמים שאת/ה לא מסוגל להשלים דבר? | 36. |
|  |  |  |  | האם את/ה מרגיש/ה לפעמים שיש לך מעט מאוד תחביבים או תחומי עניין? | 37. |
|  |  |  |  | האם את/ה מרגיש/ה לפעמים אשם/ה? | 38. |
|  |  |  |  | האם את/ה מרגיש/ה לפעמים כמו כישלון? | 39. |
|  |  |  |  | האם את/ה מרגיש/ה לפעמים מתוח/ה? | 40. |
|  |  |  |  | האם את/ה מרגיש/ה לפעמים כאילו שכפיל החליף מישהו מבני משפחתך, חבריך או מכריך? | 41. |
|  |  |  |  | האם קורה שאת/ה רואה חפצים, אנשים או בעלי חיים, שאף אחד אחר אינו רואה? | .42 |

**Scoring of the CAPE-42**

Before calculating scores, each item has to be recoded to 1-4 (לעולם לא = 1, לעיתים = 2, לעיתים קרובות = 3, כמעט כל הזמן = 4).

The overall score can be calculated by summing the scores of all items.

As suggested by Stefanis et al. (2002), the score of the original dimensions can be calculated by adding up the scores of the corresponding items.

The **positive** dimension score corresponds to the sum of the following items:

2, 5, 6, 7, 10, 11, 13, 15, 17, 20, 22, 24, 26, 28, 30, 31, 33, 34, 41, 42.

The **negative** dimension sore corresponds to the sum of the following items:

3, 4, 8, 16, 18, 21, 23, 25, 27, 29, 32, 35, 36, 37.

The **depressive** dimension score corresponds to the sum of the following items:

1, 9, 12, 14, 19, 38, 39, 40.

As suggested by Fekih-Romdhane et al. (2023), the scores for the eight subdimensions can be calculated by adding up the scores of the corresponding items.

The **depressive** dimension: as mentioned previously (items: 1, 9, 12, 14, 19, 38, 39, 40).

Within the **negative** dimension:

The **social withdrawal** subdimension corresponds to the sum of the following items:

3, 4, 29.

The **affective flattening** subdimension corresponds to the sum of the following items:

8, 27, 32.

The **avolition** subdimension corresponds to the sum of the following items:

16, 37, 18, 21, 23, 25, 35, 36.

Within the **positive** dimension:

The **bizarre experience** subdimension corresponds to the sum of the following items:

17, 24, 26, 28, 30, 31.

The **perceptual abnormalities** subdimension corresponds to the sum of the following items:

33, 34, 42.

The **persecutory ideation** subdimension corresponds to the sum of the following items:

2, 5, 6, 7, 10, 22, 41.

The **magical thinking** subdimension corresponds to the sum of the following items:

11, 13, 15, 20.

**References**

Fekih-Romdhane, Feten, Nour Farah, Diana Malaeb, Majda Cheour, Sahar Obeid, and Souheil Hallit. 2023. “Validation of the Arabic Version of the Community Assessment of Psychic Experiences (CAPE-42) in a Large Sample of Young Adults from the General Population.” *International Journal of Mental Health and Addiction*, 1–18. https://doi.org/10.1007/s11469-023-01011-3.

Stefanis, N. C., M. Hanssen, N. K. Smirnis, D. A. Avramopoulos, I. K. Evdokimidis, C. N. Stefanis, H. Verdoux, and J. Van Os. 2002. “Evidence That Three Dimensions of Psychosis Have a Distribution in the General Population.” *Psychological Medicine* 32 (2): 347–58. https://doi.org/10.1017/S0033291701005141.
